# Validation of the Achievement Emotions Questionnaire for Physical Education (AEQ-PE)

**DOI:** 10.3390/ijerph17124560

**Published:** 2020-06-24

**Authors:** Sebastián Fierro-Suero, Bartolomé J. Almagro, Pedro Sáenz-López

**Affiliations:** Faculty of Education, Psychology and Sport Sciences, Universidad de Huelva; Avda. Tres de marzo s/n, 21071 Huelva, Spain; almagro@dempc.uhu.es

**Keywords:** psychometric properties, control-value theory, basic psychological needs, adolescents

## Abstract

The fundamental role of emotions in education has been revealed in recent years. The control-value theory of achievement emotions has been postulated as one of the most used theories in this field. Thanks to the Achievement Emotions Questionnaire (AEQ), achievement emotions have been measured in different subjects and countries. The purpose of this research was to adapt and validate this questionnaire to assess achievement emotions in physical education. The sample of participants consisted of 902 (Mage = 13.15, SD = 1.17) secondary education students from various secondary schools in Spain. The psychometric properties of the Achievement Emotions Questionnaire for Physical Education (AEQ-PE) indicate that the scales are reliable and valid, as demonstrated by exploratory and confirmatory factor analysis, temporal stability, internal consistency and regression analysis. Considering the results achieved in the present study, the AEQ-PE opens a range of possibilities for both teachers and researchers. This instrument will help to understand the role of emotions in student learning and their motivation towards physical education.

## 1. Introduction

Emotions include sets of coordinated psychological processes, including affective, cognitive, physiological, motivational and expressive components [1]. Emotional experiences are always present and significant; therefore, researchers agree that emotions play a fundamental role in the learning process, affecting student motivation and performance [2,3,4,5]. For example, students may be excited while studying, hope for success, be proud of their achievements, be surprised to discover a new solution, be anxious about failing tests, be ashamed of poor grades or be bored during lessons [4]. In addition, the UNESCO, in Pekrun’s words, established that the emotional health of students should be recognized as an important educational goal in itself [6]. Therefore, teachers need to know and deal with the emotions that students experience in school [4]. 

For most students, positive emotions will be beneficial and negative emotions will be detrimental to academic learning [7]. On the one hand, learning experiences that stimulate positive emotions because they are fun and stimulating help students to have feelings of flexibility, attention, physical health and higher performance [8,9]. On the other hand, teachers who use monotonous teaching styles or create learning environments with excessive student failure will promote negative emotions such as boredom [10], anxiety and anger [11]. These negative emotions were associated with harm to student attention, reduction in interest and the promotion of superficial levels of learning and motivation [8]. In conclusion, increasing positive emotions and reducing negative emotions is crucial for all teachers in any subject [2,4,12]. Creating environments and tasks in which students can choose between different options, making time limits more flexible or giving them second opportunities have proven to be good strategies [13]. For example, students will enjoy learning if they feel competent to meet the demands of the learning task and value the learning material. On the contrary, if they feel incompetent, or are not interested in the material, learning is not enjoyable. In this case, the excessive difficulty of the task generates anger and anxiety, or the lack of intrinsic incentive value causes boredom [13]. Therefore, there is a clear relationship between emotions and motivation [14,15]. The self-determination theory (SDT) [16,17] postulates that motivation is influenced by three basic psychological needs (BPNs), which are innate and universal in all people. Autonomy refers to the feeling of being the origin of one’s actions, competence refers to feeling effective in the actions performed and relatedness refers to feeling significantly connected to others [16]. In recent years, novelty has been proposed as a possible fourth BPN (e.g., [18,19,20]) and has been defined as the need to experience something that has not been experienced before or that differs from a person´s daily routine [18]. Experiences that satisfy BPNs generate positive emotions and psychological well-being which are related to intrinsic motivation; on the contrary, frustration generates negative emotions and demotivation [21,22].

As Simonton stated [23], physical education (PE) should not be defined by a particular curriculum or teaching style, but rather by the ability to emotionally impact students. The importance of emotions in PE has garnered attention in recent years from various perspectives (e.g., [12,24]). Some of the topics studied so far have included the role of emotions in engagement and participation during classes and even in leisure-time [25,26,27]. Other research has focused on the feeling of students in various specific situations, such as learning new motor skills [28], taking a shower in school [29] or being picked in the last round for a team [30]. A large number of studies take into account some emotions or related aspects (e.g., [30,31,32,33,34]), highlighting the importance of positive emotions in facilitating learning [35,36]. The relationship between BPNs and emotions has been shown in PE, demonstrating that autonomy support helps to generate positive emotions and control tends to generate negative emotions (e.g., [12,15,37]). Emotions can provide a window into an understanding of students’ experiences and behaviors in PE [11]. For example, happiness unleashes creativity and the desire to play [4]. In spite of this importance, there is disagreement when researchers want to measure emotions, which is due to the lack of validated instruments. So far, most studies have used a more global approach by measuring the affect (positive and negative) (e.g., [38,39]). This means that they do not take into account various discrete emotions [40], and it limits the capacity to relate emotions to specific behaviors [23,40,41], which may be problematic [11]. Recently, Trigueros et al. [24] have proposed an instrument to measure specific emotions created by a Delphi method based on a review of existing instruments. Nevertheless, the results obtained are difficult to compare since they are not based on any specific theory.

Few educational theories focus on student emotions in PE, in spite of their importance [11]. In recent years, the use of the control-value theory of achievement emotions (CVTAE) in PE has been proposed [11,23,42,43,44]. Achievement emotions can be defined as emotions that are tied directly to achievement activities (e.g., studying) or achievement outcomes (success and failure) [13]. Therefore, the CVTAE offers a taxonomy in the academic field based on a social cognitive perspective [2]. Specifically, the CVTAE organizes emotions in three dimensions, based on the control and value appraisals: valence (positive or negative), activity level (activating or deactivating) and object focus (activity or outcome) (Table 1). It is important to note that CVTAE does not mean that achievement emotions are always mediated by conscious evaluations, but these can be automated with repetition [13].

This theory allowed the Achievement Emotions Questionnaire (AEQ) [45] to be developed. This questionnaire split a large number of emotions into four quadrants based on the criteria mentioned above (Table 1): (A) positive and activating emotions, namely enjoyment, hope and pride; (B) positive and deactivating emotions, namely relief; (C) negative and activating emotions, namely anger, anxiety and shame; (D) negative and deactivating emotions, namely hopelessness and boredom. The original version had 231 items which focused on three contexts: class attendance, learning and test taking. This first version has resulted in different, shorter versions, focusing attention on the most well-founded emotions to date. Moreover, the AEQ has been adapted for various subjects (e.g., mathematics [46,47], language (AEQ-L) [48], physics [49]) and has been translated into many languages (e.g., Chinese [46], Argentine Spanish [50], Korean [51]).

Recently, Simonton [11] has proposed exploring achievement emotions in PE, creating a coherent review of the use of this theory in PE. At the practical level, it has produced promising results [23,44]; however, these studies have two main problems when used to measure emotions. They have focused only on certain specific emotions [42,43,52] or they have used the class-related subscales without adapting them to PE [23,44]. Nonetheless, the usual situations in PE are different from those in the classroom. PE is characterized by being eminently practical; despite this, a recent qualitative study found that students tend to express emotions similar to those collected by the AEQ [12]. Simonton [11] highlights the importance of addressing the construction of instruments to measure emotions in PE. For these reasons, based on the satisfactory results obtained in other subjects by AEQ and the preview results obtained in PE, the aim of this study was to adapt and validate the Achievement Emotions Questionnaire for Physical Education (AEQ-PE). In addition, to contribute to its external validity, a secondary objective of studying the relationship between BPNs and the emotions measured by the AEQ-PE was established. This must be a first step, which will help to provide information about the set of emotions and understand the behaviors of students in PE [11].

## 2. Methods

### 2.1. Participants

The participants in this study were 902 secondary education students (427 males and 475 females) aged 11 to 17 years (M = 13.15, SD = 1.17) from five educational institutions (public and private) in the province of Huelva (Spain). There were students from the four grades of lower secondary education: 285 first graders, 332 second graders, 224 third graders and 61 fourth graders. The selection of the sample was non-probabilistic, since it was made depending on those secondary schools that agreed to participate in the study. To carry out the factor analyses, the sample was randomly divided into groups of approximately 50% (Table 2).

### 2.2. Measures

#### 2.2.1. Achievement Emotions Questionnaire for Physical Education (AEQ-PE)

The AEQ-PE was adapted to the AEQ-PA [47] as described in the procedure. This instrument comprises 24 items divided into six emotions (four for each emotion) covering the three main quadrants of the CVTAE [2]. These emotions are: boredom (e.g., “The physical education class bores me”), hopelessness (e.g., “I have lost all hope of doing physical education activities effectively”), anger (e.g., “I feel anger welling up in me during the physical education class”), anxiety (e.g., “I get scared that I might say/do something wrong in the physical education class, and I would rather not say/do anything”), enjoyment (e.g., “I feel good when I am in physical education class, practicing what the teacher suggests”) and pride (e.g., “I am proud of my participation in physical education class”). Responses were provided on 5-point scales (1 = totally disagree and 5 = totally agree).

#### 2.2.2. Basic Psychological Needs Satisfaction

The Spanish version, adapted to PE [53], of the Basic Psychological Needs in Exercise Scale [54], including items to measure novelty [18,55], was used. This instrument consists of four items for autonomy, competence and relatedness, and five items for novelty, all of them preceded by the stem “In my Physical Education class…”. Example items are “I feel very strongly that I have the opportunity to make choices about the way I exercise” (autonomy), “I feel that exercise is an activity in which I do very well” (competence), “I feel very much at ease with the other exercise participants” (relatedness) and “I frequently feel there are novelties for me” (novelty). Responses were provided on 5-point scales (1 = totally disagree and 5 = totally agree).

### 2.3. Procedure

The study was approved by the Andalusian Ethics Committee of Biomedical Research (TD-OCME-2018) and was carried out in accordance with the ethical principles of the American Psychological Association [56]. First, a bibliography search was conducted to find specific research to assess aspects of student emotional involvement. The CVTAE was selected because it was postulated as a consolidated theory [2]. Moreover, the AEQ has been tested in various subjects and languages and has recently been proposed in physical education. Specifically, following the latest validated versions of this instrument, enjoyment, pride, anger, anxiety, hopelessness and boredom were selected. These emotions were shown to be the most relevant and cover the three main quadrants of the CVTAE [2]. Specifically, the version for adolescents adapted to mathematics [48] was used, adapted to physical education. For this purpose, first, two translators translated the scale into Spanish; later, two other translators carried out the translation back into the original language. After that, the original and final versions were compared to check that both scales said the same thing. This was followed by the creation of an expert group of professionals in sport and psychology to adapt the necessary items to the context of physical education. Due to the peculiarity of PE, it was decided to adapt only the version referring to the “in-class” context. Thus, a shorter instrument, better adjusted to the nature of PE would be obtained. Finally, an evaluation of each of the proposals was made, and the items with higher content validity were retained for the final version of the Achievement Emotions Questionnaire for Physical Education (AEQ-PE) (see Appendix A).

Once the new scale was defined, participation in the study was requested through school administrators and school boards. Since the students were minors, the Ethics Committee required written informed consent and parental authorization for the participation. Before administering the questionnaire to the entire sample, a pilot sample of 45 students completed the questionnaire. None of them expressed comprehension problems with the items. The data were collected during school hours, with the presence of a member of the research group to answer questions [56]. The students participated voluntarily and took approximately 20 min to complete the scale.

### 2.4. Data Analysis

The psychometric properties of the AEQ-PE scale were tested to determine its validity and reliability for physical education. Because of the changes produced by the adaptation of the questionnaire to physical education and the lack of studies with prior exploratory analyses, it was considered necessary to test the instrument by an exploratory analysis. The robust unweighted least squares (RULS) method was used with promax rotation. Multiple criteria were used for factor selection [57]. Secondly, the factor structure was confirmed by confirmatory factor analysis using the maximum likelihood estimation method because there was an indication of multivariate non-normality in the data (the standardized Mardia’s kurtosis statistic was 87.54) [58]. The currently most recommended adjustment indices were used to evaluate the proposed models [59]: χ^2^/df, comparative fit index (CFI), Tucker–Lewis index (TLI), goodness of fit index (GFI), incremental fit index (IFI), root-mean-square error of approximation (RMSEA) and standardized root-mean-square residual (SRMR). Values equal to or greater than 0.90 for CFI, TLI, GFI and IFI; lower than 3 for χ^2^/df; and lower than 0.06 for RMSEA and SRMR are considered good fit indices [60,61].

On the other hand, temporal stability and internal consistency were studied using Cronbach’s alpha. Finally, a regression analysis was performed in which the BPNs acted as predictors of achievement emotions. The Factor 10.10.01, SPSS 23.0 and AMOS 23.0 (IBM, Armonk, NY, USA) packages were used for the analyses.

## 3. Results

### 3.1. Exploratory Factor Analysis

Before performing the exploratory factor analysis, the Kaiser–Meyer–Olkin test (KMO = 0.93) and Bartlett’s statistic indices (χ^2^ (276) = 4987, *p* < 0.001)) were calculated. These results show the suitability of the data for the analysis. Exploratory factor analysis (EFA) with robust unweighted least squares (RULS) estimation and promax rotation was employed to identify the latent dimensions that underlie the data. Following objective criteria [62] after comparing different models and taking into account that the AEQ questionnaire was designed to measure six emotions, it was proposed that one factor be used for each emotion (Table 3). Model fit indices offered by the Factor program [63] such as GFI (0.99) and RMSEA (0.00) show a very good fit [59,61].

### 3.2. Confirmatory Factor Analysis

**Figure 1:**
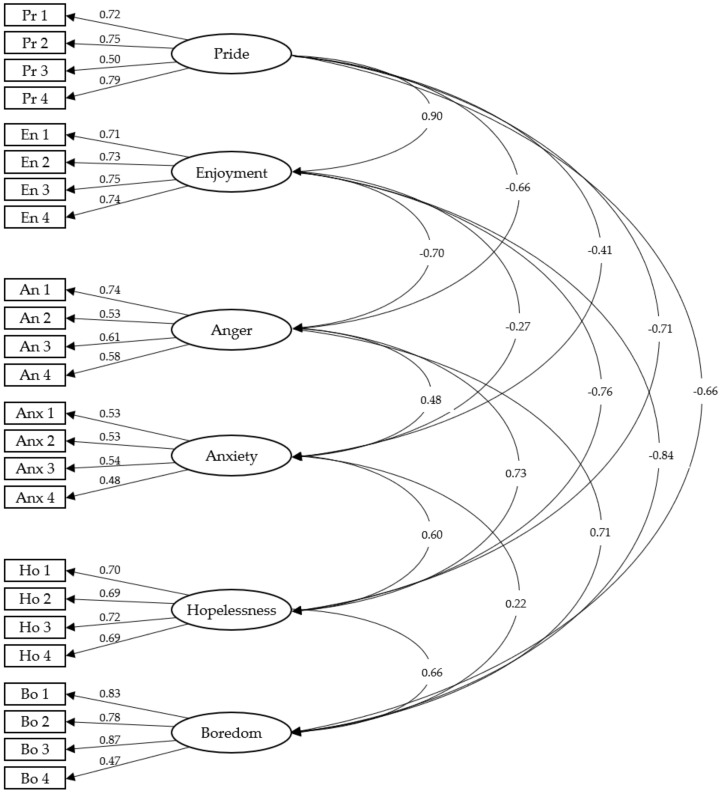
Confirmatory factor analysis of the Achievement Emotions Questionnaire for Physical Education (AEQ-PE). Pr, pride; En, enjoyment; An, anger; Anx, anxiety; Ho, hopelessness; Bo, boredom. The ellipses represent the factors and the rectangles represent the specific items.

The maximum likelihood estimation method was used in the AFC to examine the six-factor structure of the model. The results of the analysis show a good fit to the six-factor model: χ^2^ (237) = 491, *p* < 0.001, χ^2^/df = 2.072, CFI = 0.942, TLI = 0.932, IFI = 0.942, GFI = 0.915, RMSEA = 0.049, SRMR = 0.044. The standardized factor loadings were statistically significant (*p* < 0.001) and ranged from 0.47 to 0.83 (Figure 1).

### 3.3. Assessment of Internal Consistency and Temporal Stability

To measure the internal consistency of the AEQ-PE, Cronbach’s alpha was calculated (Table 4). A sample of 30 students was used to measure the temporal stability with a 30-day difference between the two measurements. Furthermore, the relationships between the different variables were studied, showing negative relationships between negative emotions (boredom, hopelessness, anger and anxiety) and positive emotions (enjoyment and pride), all of which were statistically significant.

### 3.4. Criteria Validity Analysis

To test the relationship of the AEQ-PE dimensions with other variables, a regression analysis should be performed. To this end, BPNs (including novelty) were introduced as predictors of the achievement emotions. The results (Table 5) show how BPNs have a high predictive power for emotions with significant regression weight and provided a high explained variance. The emotions with higher regression weights were positive (enjoyment and pride), and those with lower weights were negative (anger and anxiety).

## 4. Discussion

The aim of this study was to adapt and validate the AEQ to the context of Spanish PE classes for secondary school students. To this end, to normalize the scales for practical application [7], due to the peculiarity of PE, the original AEQ [45] needed to be adapted. For this adaptation, the AEQ-PA [47] was selected for several reasons. First, this scale focuses on a specific subject (mathematics) in adolescent students. Second, the AEQ-PA measures the six emotions that were shown to be the most important (enjoyment, pride, anger, anxiety, hopelessness and boredom), covering the three main quadrants of the CVTAE [2]. With this in mind the Achievement Emotions Questionnaire for Physical Education (AEQ-PE) was created, including only the versions of questions referring to the "in-class" context, which is better suited to the nature of PE. The psychometric properties of the AEQ-PE have been examined using various tests, such as exploratory factor analysis, confirmatory factor analysis, assessment of internal consistency, temporal stability and regression analysis.

Due to changes from the adaptation of the questionnaire to physical education and the lack of exploratory studies, an exploratory analysis was made. The results showed the existence of one factor for each emotion, which was confirmed by confirmatory analysis. Factor loadings were high and fit indices were good in both cases [59,60,61]. These results support the model of the emotions being differentiated, discrete and separately probeable [49]. Therefore, separation into discrete emotions is a better model than models with a single factor or with positive and negative emotions [7,47,49]. For the internal consistency, Cronbach’s alpha was accepted, as was temporal stability [64]. Positive emotions (enjoyment and pride) were highly positively related to each other and negatively related to negative emotions (boredom, hopelessness, anger, anxiety; in this order from highest to lowest correlation), all of these correlations being highly significant. The pattern of relationships between emotions is similar to those found in previous studies [7,47,49,50].

Based on the fact that the satisfaction of the BPNs generates positive emotions and their frustration generates negative emotions [21,22], a regression analysis was performed in which the BPNs acted as predictors of achievement emotions to provide external evidence. The results showed high percentages of explained variance, which were higher in positive emotions (enjoyment 51% and pride 46%) than in negative emotions (boredom 35%, hopelessness 34%, anger 18% and anxiety 16%). The strong relationship between both variables in PE has been shown recently, but not the predictive power [15], as it previously was in general learning [22]. Specifically, the psychological needs that gave greater regression weight were competence and novelty in both the positive and negative emotions. The analysis of the regression weights suggests how specific strategies to generate positive emotions and avoid the generation of negative emotions during the physical education class may be established. In this case, as in previous studies, anxiety was the emotion that was least correlated with the other emotions and had lower regression weights, because anxiety has complex effects on students [49]. Despite this, all the correlations were statistically significant, and the explained variances were considerable.

The results of the present study provide psychometric support for the AEQ-PE. Nevertheless, some limitations need to be considered. First, this study was carried out with a convenience sample of Spanish pre-adolescents in PE; in the future, other similar groups of students should be examined. Second, the level of measurement invariance has not been studied. For that reason, future research should take this into account when considering that the instrument validation is a continuous process. Similarily, it is recommended that future research continues to study the relationship between the BPNs and other motivational variables with emotions, as well as to find strategies to generate positive emotions and avoid negative emotions in PE. These strategies should be in harmony with the satisfaction of the BPNs as proposed by Deci and Ryan [21]; however, this needs more attention [15].

## 5. Conclusions

In conclusion, the results of this study provide support for the validity and reliability of the Achievement Emotional Questionnaire for Physical Education (AEQ-PE). This instrument has been shown to measure the main emotions of the CVTAE [2] with satisfactory psychometric properties. The CVTAE [2] is a theory with broad scientific support and application in education, which makes it possible to compare results between different subjects or areas (e.g., [46,48,49,50]). Moreover, the AEQ-PE helps to obtain different emotion profiles experienced by students in practical PE situations. This information is of great interest to teachers to establish strategies that generate positive emotions and avoid negative emotions, because they need to customize their practices to meet individual needs [13]. It is equally interesting for researchers, as it will allow the studying of the role that emotions play in academic performance, student participation, motivation for physical activity, etc. Therefore, the AEQ-PE opens a range of possibilities for the study of emotions in PE.

## Figures and Tables

**Table 1:** Example of achievement emotions classified by valence, activation and object focus.

Object Focus	Positive		Negative	
	Activating	Deactivating	Activating	Deactivating
Activity focus	Enjoyment	Relaxation	Anger	Boredom
Outcome focus	Pride	Relief	Anxiety	Hopelessness

**Table 2:** Sample distribution for factor analysis.

Factor Analysis	*N*	Males	Females	Aged
Exploratory Factor Analysis (EFA)	443	218 (49.2%)	224 (50.6%)	13.15 (1.21)
Confirmatory Factor Analysis (CFA)	358	204 (45.2%)	246 (54.5%)	13.14 (1.21)

**Table 3:** Exploratory factor analysis.

Factors and Items	1	2	3	4	5	6
**Factor 1: Pride**						
I am proud to be able to keep up with the physical education class.	0.74					
I am proud of my participation in physical education class.	0.71					
I think that I can be proud of what I know about physical education.	0.65					
Because I take pride in my accomplishments in physical education, I am motivated to continue.	0.78					
**Factor 2: Enjoyment**						
I am motivated to go to the physical education class because it is exciting.		0.73				
I enjoy being in the physical education class.		0.71				
I feel excited about being in physical education class, practicing what the teacher suggests.		0.58				
I am glad going to the physical education class paid off.		0.51				
**Factor 3: Anger**						
I feel anger welling up in me during the physical education class.			0.68			
Because I am angry, I get restless in the physical education class.			0.47			
Thinking about all the useless things I have to learn in physical education, annoys me.			0.57			
After the physical education class, I am angry.			0.80			
**Factor 4: Anxiety**						
I worry that the things I have to do in physical education classes might be too difficult.				0.60		
I feel nervous in the physical education class.				0.58		
I get scared that I might say/do something wrong in the physical education class, and I would rather not say/do anything.				0.59		
When I do not understand something in the physical education class, my heart races.				0.59		
**Factor 5: Hopelessness**						
It is pointless to prepare for the physical education class because I am bad at it anyway.					0.73	
Even before entering the physical education class, I know I will not get it right.					0.62	
I would rather not go to the physical education class because it is impossible to perform the exercises correctly.					0.58	
I have lost all hope of doing physical education activities effectively.					0.46	
**Factor 6: Boredom**						
I feel like leaving during the physical education class because it is so boring.						0.74
I get bored during the physical education class.						0.82
The physical education class bores me.						0.98
I find the physical education class fairly dull.						0.53

Note: Factor loadings less than 0.4 are not shown in the table. The rotation converged in 10 iterations.

**Table 4:** Descriptive statistics, internal consistency, temporal stability and convergent validity.

Emotions	*M*	*SD*	α	ICC	1	2	3	4	5	6
1. Pride	3.95	0.89	0.78	0.76	-	0.71	−0.44	−0.26	−0.52	−0.52
2. Enjoyment	4.00	0.94	0.83	0.70		-	−0.50	−0.24	−0.58	−0.71
3. Anger	1.50	0.72	0.73	0.68			-	0.34	0.55	0.58
4. Anxiety	2.00	0.87	0.72	0.70				-	0.45	0.20
5. Hopelessness	1.48	0.72	0.79	0.71					-	0.51
6. Boredom	1.88	0.92	0.82	0.72						-

Notes: α, Cronbach’s alpha; *M*, mean; *SD*, standard deviation; ICC, intra-class correlation coefficient. All correlations were significant at *p* < 0.001.

**Table 5:** Results of regression analyses.

Variables	R^2^	*Β*	T	*P*
**Pride**	0.46			
Autonomy		0.06	1.56	0.12
Competence		0.48	14.22	0.00
Relatedness		0.06	2.01	0.05
Novelty		0.20	5.91	0.00
**Enjoyment**	0.51			
Autonomy		0.17	4.66	0.00
Competence		0.34	10.23	0.00
Relatedness		0.09	2.86	0.00
Novelty		0.31	8.95	0.00
**Anger**	0.18			
Autonomy		−0.05	−1.56	0.12
Competence		−0.15	−4.65	0.00
Relatedness		−0.14	−4.67	0.00
Novelty		−0.08	−2.45	0.01
**Anxiety**	0.16			
Autonomy		−0.00	−0.10	0.92
Competence		−0.29	−7.14	0.00
Relatedness		−0.22	−5.93	0.00
Novelty		0.14	3.40	0.00
**Hopelessness**	0.34			
Autonomy		0.03	1.03	0.30
Competence		−0.42	−13.98	0.00
Relatedness		−0.06	−2.07	0.04
Novelty		−0.07	−2.43	0.02
**Boredom**	0.35			
Autonomy		−0.15	−3.84	0.00
Competence		−0.12	−3.11	0.00
Relatedness		−0.09	−2.61	0.01
Novelty		−0.35	−9.24	0.00

## Appendix A. Achievement Emotions Questionnaire for Physical Education (AEQ-PE)

**Table A1:** This questionnaire was validated in Spanish.

1. No tiene sentido prepararme para la clase de Educación Física porque, de todos modos, se me da mal.
2. Incluso antes de entrar a clase de Educación Física, ya sé que no lo conseguiré hacer bien.
3. Me motiva ir a clase de Educación Física porque es emocionante.
4. Me preocupa la dificultad de las cosas que podrían pedirme hacer en clase de Educación Física.
5. Preferiría no ir a clase de Educación Física porque, de todos modos, es imposible realizar los ejercicios correctamente.
6. Disfruto estando en clases de Educación Física.
7. Tengo ganas de que termine la clase de Educación Física porque es muy aburrida.
8. Me enorgullece ser capaz de seguir el ritmo de la clase de Educación Física.
9. Me aburro durante la clase de Educación Física.
10. Siento un enfado que va creciendo en mi interior durante la clase de Educación Física.
11. Me siento bien cuando estoy en clase de Educación Física practicando lo que propone el profesor/a.
12. Me siento nervioso/a en clase de Educación Física.
13. Me aburre la clase de Educación Física.
14. Estoy orgulloso/a de mi participación en clase de Educación Física.
15. Debido a que estoy enfadado/a en clase de Educación Física, me siento impaciente.
16. He perdido toda esperanza de hacer eficazmente las actividades de Educación Física.
17. Me da miedo poder decir/hacer algo incorrecto en clase de Educación Física, preferiría no decir/hacer nada.
18. Me irrita pensar en todas las cosas inútiles que tengo que aprender en Educación Física.
19. La clase de Educación Física me parece bastante monótona.
20. Cuando no entiendo algo en clase de Educación Física se me acelera el corazón.
21. Me siento enfadado/a después de la clase de Educación Física.
22. Creo que puedo estar orgulloso/a de mis conocimientos de Educación Física.
23. Estoy contento/a de que valga la pena ir a clase de Educación Física.
24. Como me siento orgulloso/a de mis logros en Educación Física, estoy motivado/a para continuar.
